# Pharmacotherapy for Essential Hypertension: A Brief Review

**DOI:** 10.14797/mdcvj.1175

**Published:** 2022-12-06

**Authors:** Behnam Heidari, Eleonora Avenatti, Khurram Nasir

**Affiliations:** 1 Houston Methodist DeBakey Heart & Vascular Center, Houston Methodist, Houston, Texas, US

**Keywords:** essential hypertension, pharmacotherapy, calcium channel blockers, beta blockers, diuretics, angiotensin receptor blockers, angiotensin converting enzyme inhibitors

## Abstract

Hypertension is one of the leading causes of disability-adjusted life years and mortality, with approximately 15% prevalence worldwide. Most patients with hypertension from low- to high-income countries do not receive treatment. Among those who receive treatment, the majority remain undertreated and do not achieve their blood pressure goals. Therefore, new hypertension guidelines introduce more conscientious treatment strategies to maximize the probability of achieving the new strict blood pressure goals compared with the previous guidelines. Who should receive treatment for hypertension? Which antihypertensive medications have the strongest supporting data? Are generic and more affordable medications as effective as expensive brand medications? What are the different treatment strategies to maximize success in controlling blood pressure? Here, we briefly review pharmacotherapy for hypertension and provide answers to these questions as well as some other common questions regarding treatment of hypertension.

## Introduction

In 2019, approximately 1.2 billion people globally were estimated to have hypertension—twice as many as the year 1990.^1^ Hypertension is one of the leading causes of mortality and disability-adjusted life years worldwide,^2,3^ and it remains one of the most important modifiable contributing factors to the burden of coronary artery disease, stroke, and chronic kidney disease.^2,3,4^ Studies have shown that the risk of fatal cardiovascular events doubles for each 20-mm Hg increase in systolic or 10-mm Hg increase in diastolic blood pressure (BP).^5^ Hypertension can be easily diagnosed, and a wide variety of inexpensive therapies are available to effectively control it.^1,6^

**Figure 1:**
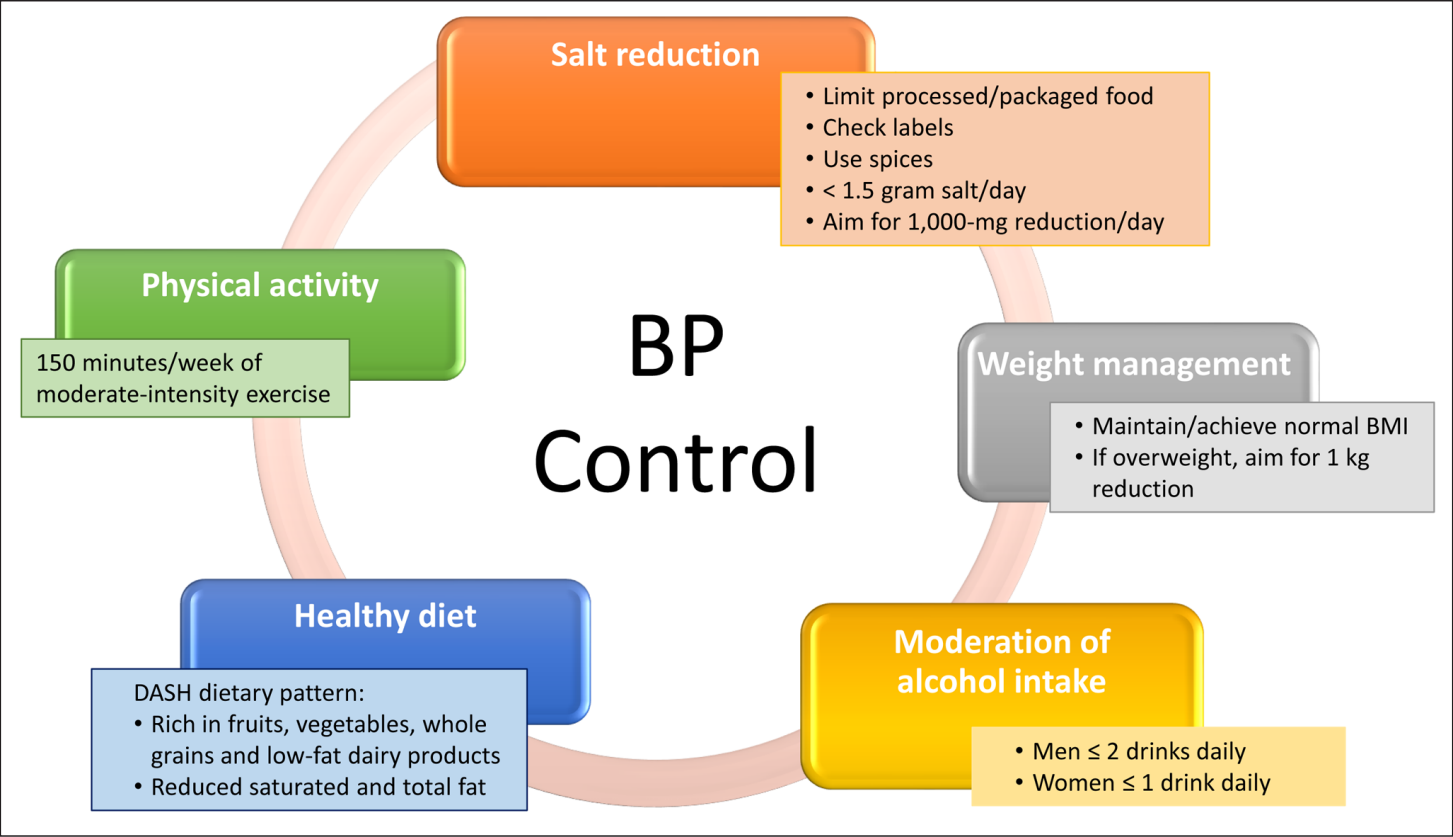
Non-pharmacological approaches to hypertension treatment. Attention to diet, salt and alcohol reduction, and physical activity need to be part of the therapeutic approach for all patients.

Controlling hypertension is associated with a reduction in mortality and adverse cardiovascular outcomes,^7^ and both non-pharmacological and pharmacological interventions are essential to treatment.^8^ Non-pharmacological interventions include reducing dietary sodium, increasing consumption of fruits and vegetables,^9,10^ a high-protein low-carbohydrate diet,^11^ and losing weight,^12^ all of which should be included throughout the treatment period (Figure 1).

Pharmacological interventions were first established in the late 1950s with thiazide diuretics, the first class of medications to be studied in a clinical trial and the first to be used for treating hypertension.^13,14^ Following the favorable outcome with thiazide diuretics, several other studies were conducted and showed the efficacy of other BP-lowering medications in controlling hypertension and preventing related complications.^15,16,17,18,19,20,21,22,23,24^ The landmark Treatment of Mild Hypertension Study—the first to compare the efficacy of different classes of BP-lowering medications—showed that various antihypertensive medications have considerable effect in reducing BP with minimal differences among different classes.^25^ We still use most of these established BP medications to treat hypertension in our daily medical practice based on the findings of these landmark studies.^8^ In this article, we discuss pharmacologic management of essential hypertension and briefly review the indications and use of different medication classes.

## Defining Hypertension and When to Start Pharmacotherapy

**Figure 2:**
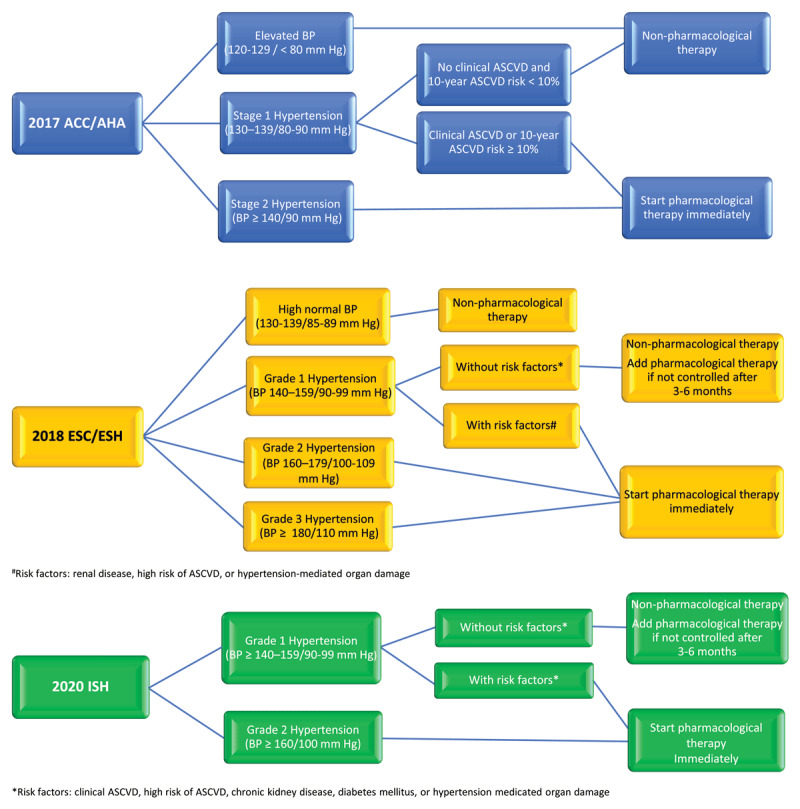
Overview of recommendations for hypertension treatment by current international guidelines. ACC/AHA: American College of Cardiology/American Heart Association; ESC/ESH: European Society of Cardiology/European Society of Hypertension; ISH: International Society of Hypertension

Although major hypertension guidelines use different BP thresholds to define hypertension and its stages, they all use a combination of BP level and patient risk factors including atherosclerotic cardiovascular disease (ASCVD) risk for their recommendations on BP management (Figure 2). The 2017 American College of Cardiology (ACC)/American Heart Association (AHA) guideline for treatment of BP defines normal BP as < 120/80 mm Hg. It recommends non-pharmacological therapy for elevated BP (120-129/< 80 mm Hg) and for stage 1 hypertension (130-139/80-90 mm Hg) without clinical ASCVD or 10-year ASCVD risk < 10%. Non-pharmacological plus pharmacological therapy is recommended for those with stage 1 hypertension with clinical ASCVD or 10-year ASCVD risk ≥ 10% and in all patients with stage 2 hypertension (BP ≥ 140/90 mm Hg).^8^

The 2020 International Society of Hypertension (ISH) Global Hypertension Practice Guidelines,^26^ and the 2018 European Society of Cardiology (ESC)/European Society of Hypertension (ESH) guidelines^27^ have a slightly higher threshold for pharmacotherapy of hypertension. The 2020 ISH Global Hypertension Practice Guidelines recommend immediate pharmacotherapy for patients who have grade 1 hypertension (BP ≥ 140-159/90-99 mm Hg) and clinical ASCVD, high risk of ASCVD, chronic kidney disease, diabetes mellitus, or hypertension-mediated organ damage. Pharmacotherapy is indicated for all patients who have grade 2 hypertension defined as BP ≥ 160/100 mm Hg regardless of their risk or comorbidities.^26^ The 2018 ESC/ESH guidelines recommend immediate pharmacotherapy in grade-1 hypertension (BP 140-159/90-99 mm Hg) with renal disease, high risk of ASCVD, or hypertension-mediated organ damage. Grade 2 (BP 160-179/100-109 mm Hg) and grade 3 (BP ≥ 180/110 mm Hg) hypertension need pharmacotherapy regardless of comorbidities or ASCVD risk.^27^

## Medication Class Selection

There is a general recommendation for using one of three classes of medications—thiazide diuretics, angiotensin-converting enzyme inhibitors (ACEi)/angiotensin II receptor blockers (ARBs), or calcium channel blockers (CCBs)—as the initial therapy for patients with essential hypertension.^8^ Several studies have shown that different BP-lowering medications have similar efficacy in treating hypertension and preventing its complications, with minimal differences between drug classes.^25,28,29,30,31^ A meta-analysis studying 31 randomized trials demonstrated decreased major cardiovascular events following reduction of BP. This effect remained significant when patients were divided into two age groups (< 65 years or ≥ 65 years), showing both older and younger adults will benefit from treatment of hypertension. However, after analyzing the data for patients who were treated with diuretics, ACEi, ARBs, CCBs, and beta blockers, the study did not show any significant benefit for selecting one class of antihypertensive medications over another.^29^ Another meta-analysis published a year later included 147 studies that were published between 1966 and 2007, and it analyzed the data for 464,164 patients. It showed that although beta blockers were associated with decreased coronary heart disease events if used shortly after a myocardial infarction, and CCBs were associated with a slightly lower risk of stroke, all antihypertensive medication classes were associated with a comparable reduction in coronary artery disease or stroke. Again, the result of this study confirmed the importance of controlling BP in different age groups regardless of the medication used.^30^

A recent systematic review and meta-analysis that included 123 studies and 613,815 patients showed a significant reduction in major cardiovascular events and mortality for each 10 mm Hg reduction of systolic BP. This study showed that beta blockers were inferior to the other classes in preventing such adverse events, CCBs were superior in preventing stroke and inferior in preventing heart failure, and diuretics were superior in preventing heart failure.^31^ A recent Cochrane systematic review article suggested that the use of low-dose thiazide diuretics, ACEi, or CCBs as first-line therapy have similar effects in reducing mortality and morbidity in patients with moderate-to-severe essential hypertension while beta blockers or high-dose thiazide diuretics might be less effective in reducing such outcomes.^32^ Overall, in the absence of a specific indication for an antihypertensive medication, major guidelines recommend focusing on the treatment of hypertension and not the medication class selection to achieve the goal of reducing major cardiovascular events.^8,26,27^ Table 1 shows some of the common uses and precautions for the most used antihypertensive medications.

**Table 1:** Indications and contraindications for commonly used antihypertensive medications. ACE: angiotensin-converting enzyme inhibitor; Afib/flutter: atrial fibrillation/flutter; ARB: angiotensin II receptor blockers; CCB: calcium channel blocker; CKD: chronic kidney disease; HFrEF: heart failure with reduced ejection fraction; HFpEF: heart failure with preserved ejection fraction; MDD: major depressive disorder


MEDICATION CLASS	CONSIDER USING	AVOID USING

Thiazide diuretics	Conditions associated with edema such as HFrEF and HFpEF	Hyperuricemia, hyponatremia, hypercalcemia, sulfa allergy

ACEi/ARB	HFrEF, CKD with proteinuria	Pregnancy, severe hyperkalemia, bilateral renal artery stenosis, angioedema (ACEi)

CCB	Prior stroke, stable angina, and Raynaud phenomenon (dihydropyridine), Afib/flutter and migraine headaches (non-dihydropyridine)	HFrEF (class 3 or 4), 2nd- or 3rd-degree AV nodal block, bradycardia, and sick sinus syndrome (nondihydropyridine)

Aldosterone antagonists	HFrEF, resistant hypertension	Severe hyperkalemia


## Monotherapy or Multidrug Strategy

Although previous guidelines recommended starting treatment with monotherapy with gradual dose increase—switching to another class of medication, or adding a second medication if needed—recent guidelines recommend initiating two BP-lowering drugs simultaneously in most patients.^8,26,27^ However, research shows that approximately 60% of patients with hypertension are not treated. This finding remains consistent worldwide across low- to high-income countries. Importantly, of the 40% of patients who receive treatment, approximately 65% do not achieve the target of 140/90 mm Hg.^33^ In addition, the BP goal has been decreasing in the past several years, which makes it hard for monotherapy to achieve the new stricter goals. Increasing the dose of a single medication has little incremental BP-lowering effect and can potentially increase the risk of side effects. Importantly, most patients who participated in the published clinical trials were either started on multidrug therapy or needed to be treated with more than one BP-lowering medication throughout the trial.^29,30,31,32^ Therefore, recent guidelines recommend initiating treatment with more than one BP-lowering medication in most patients with hypertension.^8,26,27^

The 2018 ESC/ESH guideline recommends that monotherapy may be sufficient in patients with high normal BP (130-139/85-89 mm Hg) if their BP is close to the threshold of 140/90 mm Hg and non-pharmacological therapy has failed to control hypertension. Otherwise, it recommends the initiation of treatment with two different classes of medications for most patients who meet criteria for treatment as discussed above.^27^ The 2020 ISH Global Hypertension Practice Guideline recommends initiating pharmacotherapy with low doses of two different classes of medications in patients with hypertension who require pharmacotherapy.^26^ The 2017 ACC/AHA guideline recommends initiation of therapy with two first-line medications from different classes in patients with stage 2 hypertension (BP ≥ 140/90 mm Hg). Monotherapy can be considered in patients with stage 1 hypertension (BP = 130-138/80-90 mm Hg) with increasing the medication dose and adding other agents if needed to achieve BP goal < 130/80 mm Hg.^8^

## Morning, Evening, or Multiple Daily Dosing

The average nighttime BP is about 15% lower than daytime BP. Non-dipping phenomenon refers to the failure of BP to decrease by at least 10% of its daytime value while sleeping at night and is associated with adverse cardiovascular events. Some studies have recommended that moving BP medication doses to the evening can be associated with decreased non-dipping phenomenon.^34,35,36,37,38^ Many other clinical trials have shown no significant difference in BP readings among patients who take BP-lowering medications in the morning or in the evening.^39,40,41^ A Cochrane systematic review included 21 randomized controlled trials and analyzed the data for 1,993 patients with hypertension. It showed that evening dosing of BP medications can be associated with better BP control compared to morning administration. However, given that none of the studies reported clinically relevant outcome measures including mortality or cardiovascular events, the significance of this better BP control remains unclear.^42^ Importantly, in the recently published TIME (Treatment In Morning versus Evening) study, 21,104 patients were randomly assigned to morning versus evening dosing of BP medications. This randomized clinical trial showed no significant difference in major cardiovascular events between the study groups and concluded that patients can take their BP-lowering medications at their convenience throughout the day.^43^ Although splitting BP-lowering medication doses to more than once a day might seem to cause less fluctuations in BP levels, this increase in the number of daily doses of medications can be associated with decreased long-term medication adherence and increased treatment failure.^44,45^ All three of the 2017 ACC/AHA, 2018 ESC/ESH, and the 2020 ISH Global Hypertension Practice Guidelines recommend once-daily dosing over multiple daily dosing to improve medication adherence and decrease the risk of treatment failure. They also recommend a single pill strategy (one pill that combines two different medications) for initial treatment of hypertension as an additional step for increasing the chance of medication adherence. However, they do not give any specific recommendations for morning or evening dosing of the medications.^8,26,27^

## Generic Versus Branded Drugs

Given the global burden of hypertension and the significant costs associated with treatment, the use of generic drugs offers potential benefits in terms of healthcare cost savings and hence availability of treatment for a wider population. Although guidelines based on the principle of bioequivalence and drug approval processes do not make any distinction in this regard,^46^ adoption of generic drugs is sometimes hindered by doubts among providers and more often patients regarding efficacy and safety.^47,48^ At the same time, studies directly comparing clinical efficacy are sparse and are not required by current regulations, which focus on biologic equivalence to then infer comparable clinical efficacy.

Meta-analyses have attempted to provide some guidance, with the caveat that often the included studies focused on determination of bioequivalence and are thus characterized by small sample size, short follow-up, and inclusion of mostly young healthy individuals. An initial large systematic review and meta-analysis of generic and brand-name drugs used to treat CV diseases (including beta blockers, diuretics, CCBs, ACEi) included 47 publications, 38 of which were randomized clinical trials, and did not find any evidence of superiority of innovator to generic drugs.^49^ A more recent meta-analysis published by Manzoli et al. in 2016 substantially confirmed the same findings and further strengthened the safety of generic medications.^50^

Large recent population datasets are in line with this conclusion. In an observational retrospective study in a dataset of 9,413,620 insured people, Ti et al. evaluated 17 branded versus generic pharmaceutical substances for the treatment of hypertension/heart failure, hyperlipidemia, and diabetes mellitus and compared the hazard ratios for all-cause death and major adverse cardiac and cardiovascular events.^51^ The study concluded that generic versions were at least similar, if not superior, to branded medications. Two recent large community-based randomized controlled trials in a Chinese population followed a total of 29,000 hypertensive patients propensity score matched for use of brand versus generic medications. The results either showed no difference in the mean reduction in systolic BP, hypertension control rate, or CV outcomes^52^ or demonstrated higher hospitalization rates for CVD in patients started with some of the branded drugs analyzed, possibly pointing to a difference in medication adherence in the brand prescription groups.^53^ Indeed, in both studies the use of generic drugs unsurprisingly translated into lower medication costs.

Finally, a small open crossover randomized controlled trial in France allocated hypertensive patients to their usual antihypertensive treatment either exclusively with brand-name drugs for 6 weeks and then switching to generics for another 6 weeks or following the reverse order.^54^ Twenty-four hour ambulatory BP monitoring demonstrated no significant impact of branded versus generic drug use (mean 24-h average BP of 129/77 vs 128/77 mm Hg for generic vs brand drugs, respectively). Taken together, these findings support the safety and efficacy of generic medications for BP treatment, with possible advantages in effectiveness given the lower economic burden on patients.

## Less Commonly Used Medications

Other drug classes can be used to lower BP, namely beta blockers, alpha 1 blockers, central acting alpha 2 blockers, and direct vasodilators. These are all considered secondary agents because long-term use was not associated with survival benefit.^8^ Their use should be considered only after exhausting other proven interventions including lifestyle changes and first-line drugs and having excluded forms of secondary hypertension. As such, these medications should be cautiously evaluated in each patient based on the presence of comorbidities and concomitant indications independent of BP control.

There are currently no data to support the use of beta blockers for the treatment of hypertension, barring the presence of specific comorbidities that represent a strong indication for beta blockade (ie, ischemic heart disease or heart failure). Data showed worse clinical outcomes with beta blockers compared to ARBs despite a similar drug-mediated reduction in BP values.^55^

The ALLHAT (Antihypertensive and Lipid-Lowering Treatment to Prevent Heart Attack) trial was initially designed as a four-drug comparison with the alpha blocker doxazosin being tested against chlorthalidone amlodipine and lisinopril. The finding of increased risk of heart failure and stroke led to the premature cessation of the doxazosin arm and a re-evaluation of the role of alpha blockers in hypertension treatment. Current guidelines thus recommend the use of alpha 1 blockers (doxazosin, prazosin, and terazosin) as possible adjunct treatment in patients with benign prostatic hypertrophy. Of note, their use is associated with significant orthostatic hypotension, especially after the first administration, which is particularly concerning in older adults; therefore, a bedtime administration is thus usually recommended.^56^

Clonidine is a centrally acting alpha 2 agonist that reduces BP levels through negative feedback on norepinephrine release. Given its mechanism of action, it is burdened by significant side effects (from peripherally mediated constipation and dry mouth to more substantial central nervous system side effects of drowsiness and sedation). Therefore, it is usually reserved as a last-line choice. Moreover, abrupt discontinuation is associated with significant rebound hypertension and tachycardia, thus discontinuation requires very gradual taper. Current guidelines^8,27^ mention its use in a very limited setting of resistant hypertension and hypertensive emergency, although safer alternatives are often available as well.

Hydralazine and minoxidil exert their antihypertensive effect through direct vascular dilatation. The resulting compensatory reflex tachycardia and water retention require these drugs to be used in combination with a beta-blocker and diuretic^8^ Moreover, specific side effects should also be considered, since hydralazine is associated with risk of drug-induced lupus and minoxidil is associated with hirsutism and risk of pericardial effusion. As such, the use of these medication has been declining, and current guidelines recommend their use in limited settings, including resistant hypertension and hypertension in pregnancy for hydralazine, although safer options are also available.^8^

## Approach to Resistant Hypertension

Resistant hypertension is defined as uncontrolled clinic BP (> 140/90 mm Hg) despite treatment with three antihypertensive medications with complementary mechanisms of action including a diuretic. Guidelines further specify that these three antihypertensive medications should include optimal doses of an ACEi or ARB, a CCB, and a diuretic.^57^ When taking care of patients with difficult-to-control hypertension, it is critical to avoid misdiagnosis and accurately exclude the presence of secondary hypertension as well as pseudo-resistance. The latter can be attributed to incorrect techniques in BP measurements, white coat hypertension, medication nonadherence or patient intolerance to certain medications, and concomitant use of drugs or substances with hypertensive effects, including a high amount of dietary sodium.

Pseudo-resistance is a significant and underdiagnosed problem; hence, it needs to be considered and excluded to allow proper identification of patients with true resistant hypertension and focused treatment efforts. Salt consumption remains high in the general population, is associated with increased BP values, and is a significant barrier in hypertension management. A small Italian study using 24-hour urinary sodium excretion in patients with suspected resistant hypertension found that only 27% of the patients were following recommendations on salt consumption.^58^ Reduction in salt consumption is associated with reduced BP, with an effect that is more evident in hypertensive patients than in normotensive controls.^10^ Likewise, adherence to prescribed medications is a major factor in obtaining and maintaining BP control and thus preventing CVD. Estimation of adherence is difficult since methods to objectively confirm it are cumbersome and costly, and many different factors play into a patient’s willingness and ability to initiate and maintain a chronic treatment regimen.^45^ Awareness of those factors and the risk of nonadherence is critical knowledge for the managing physician. These aspects of care should be emphasized in any patient-physician discussion as part of an effective hypertension management strategy.^59^

Secondary causes of hypertension (renal arterial stenosis, hyperaldosteronism, obstructive sleep apnea, pheochromocytoma, etc) should be identified because they require different treatment approaches focused at addressing the underlying disease.^8^ Of note, the relative frequency of these diseases varies significantly, as does the likelihood of complete resolution of hypertension once the underlying disease has been addressed. For example, it is reasonable to expect normalization of BP values once a rare pheochromocytoma has been surgically removed; however, in the more common scenario of a patient with obstructive sleep apnea, use of continuous positive air pressure might address the respiratory issues but the underlying comorbidities associated with such a phenotype will make continuation of hypertension treatment likely necessary.^60^

The physiopathology of true resistant hypertension is still poorly understood. However, one of the most accepted hypothesis calls into play altered sodium homeostasis and inappropriate kidney-mediated sodium retention. The PATHWAY-2 (Optimum Treatment for Drug-Resistant Hypertension) study was a double-blind placebo-controlled crossover trial that evaluated the efficacy of medications targeting this system in improving BP control in resistant hypertension, ultimately sanctioning the superiority of spironolactone over alpha and beta blockers.^61^ A total of 230 patients completed all treatment steps (12 weeks of once-daily treatment with each of spironolactone, bisoprolol, doxazosin and placebo on top of baseline BP treatment). Patients treated with spironolactone had the most BP reduction (mean reduction of 8.7 mm Hg compared to 4.03 mm Hg for doxazosin and 4.26 for bisoprolol).

Spironolactone has thus emerged as the drug of choice in this context and is currently recommended as the fourth drug to be added to the standard treatment of hypertension.^57^ This holds true specifically for patients with normal potassium levels (K < 4.5 mEq/L) based on the limited but present risk of hyperkalemia in the PATHWAY 2 trial; for patients with higher K levels, a doubling of the thiazide diuretic dose is recommended. In case of intolerance to spironolactone, other potassium-sparing diuretics might be considered, including amiloride and eplerenone. The ACC guidelines are somewhat less direct, suggesting treatment with spironolactone while maximizing diuretic dosage and adding “other agents with different mechanism of action” to obtain BP control in resistant hypertension. Regarding the choices for the latter, the PATHWAY 2 trial supports the efficacy of bisoprolol and doxazosin in improving BP control in the setting of resistant hypertension, although the reduction was to a lesser degree compared with spironolactone.^61^

Clonidine has been studied in this setting as well. In the ReHOT (Resistant Hypertension Optimal Treatment) trial, 187 patients were randomized to clonidine versus spironolactone as the fourth drug for BP control. Although inferior to spironolactone in the amount of 24-hour BP reduction in both systolic and diastolic values, clonidine achieved similar rates of BP control.^62^ As mentioned above, significant side effects still hamper the use of clonidine, and safer options are available. Some data are also available to support the use of both hydralazine and minoxidil in this clinical setting.^63^ However, they are used infrequently because of significant side effects of fluid retention and tachycardia.^27^

Device-based treatment approaches have been evaluated given the substantial body of evidence linking the autonomic nervous system activity in the physiopathology of hypertension.^64^ Renal denervation and chronic baroreceptor stimulation have been studied in randomized clinical trials with negative or conflicting results and safety concerns, and such approaches are thus not currently recommended.^65,66^

## Recent Developments

The selective sodium-glucose cotransporter-2 (SGLT2) receptor inhibitors (empagliflozin, canagliflozin, dapagliflozin) demonstrated BP-lowering effects through various mechanisms that may include natriuresis, osmotic diuresis, and reduction of the sympathetic tone.^67^ The degree of actual BP reduction was quantified in the range of 2 mm Hg to 3 mm Hg in meta-analysis evaluation of available randomized clinical trials, a modest but nevertheless sizable impact.^68^ Cardiovascular benefits of these medications are well proven, especially in the treatment and prevention of heart failure, and the additional effect on BP should be taken into consideration in devising a patient-specific strategy.^69^

Glucagon-like peptide 1 receptor agonists (GLP1-RA), revolutionary drugs in term of medical management of obesity, positively impact BP in multiple ways above and beyond the expected positive effects of weight loss on hypertension. Indeed, the reduction in BP shown in clinical trials with GLP1-RA was observed early in the treatment, before significant weight loss occurred, suggesting independent action of these medications on hypertension.^70^ The effect is sizable, with liraglutide and semaglutide demonstrating a reduction in systolic BP in the range of 3.5 to 5.6 mm Hg and 3.9 to 6.2 mm Hg, respectively, in their pivotal randomized clinical trials.^71^ The proposed mechanisms for these antihypertensive effects might include natriuresis and increased urinary output, direct vasodilation via dedicated receptors in blood vessels, decreased sympathetic activity, or improved endothelial function through resolution of negative effects of hyperglycemia.^72^

Both of these classes might represent an important ancillary medical strategy to optimize BP control in high-risk populations in which multiple comorbidities such as obesity, type 2 diabetes mellitus, and metabolic syndrome might be adequately addressed at the same time.^73^

## Conclusion

Hypertension is one of the leading causes of mortality and morbidity worldwide. Approximately 15% of the world population has hypertension, and most of these patients either do not receive any treatment or do not achieve BP target even if they are receiving treatment. Multiple studies have shown, and all major guidelines consistently recommend, that overall, the magnitude of BP reduction and not the use of a specific group of antihypertensive medication class is the major determinant of reducing adverse cardiovascular events. The use of multiple drug therapy as the initial approach and strategies that can improve patient compliance—including one-time dosing, the use of generic and more affordable medications, or using the medication that is well tolerated—can be associated with more consistent BP control and therefore more significant reduction in future events.

## Key Points

- Non-pharmacological intervention (salt reduction, weight management, appropriate diet, physical exercise, limitation of alcohol intake) are key strategies for hypertension treatment and should be implemented throughout the course of treatment for this patient population.
- First-line treatment agents include thiazide diuretics, angiotensin-converting enzyme inhibitors/angiotensin II receptor blockers, and calcium channel blockers.
- Combination treatment with two drugs from different classes is favored as initial treatment when pharmacotherapy is considered.
- There are no data to support the use of branded versus generic drugs or indicating a preferential timing for drug administration. Once-daily medication and combination pills might improve adherence.
- In difficult-to-control hypertension, pseudoresistance should be evaluated and addressed and secondary causes of hypertension should be considered; when a fourth drug is needed, spironolactone is the drug of choice.

## CME Credit Opportunity

Houston Methodist is accredited by the Accreditation Council for Continuing Medical Education (ACCME) to provide continuing medical education for physicians.

Houston Methodist designates this Journal-based CME activity for a maximum of *1 AMA PRA Category 1 Credit™*. Physicians should claim only the credit commensurate with the extent of their participation in the activity.

Click to earn CME credit: learn.houstonmethodist.org/MDCVJ-18.5.
